# Impaired cerebral blood flow and oxygenation during exercise in type 2 diabetic patients

**DOI:** 10.14814/phy2.12430

**Published:** 2015-06-24

**Authors:** Yu-Sok Kim, Thomas Seifert, Patrice Brassard, Peter Rasmussen, Allan Vaag, Henning B Nielsen, Niels H Secher, Johannes J van Lieshout

**Affiliations:** 1 Department of Internal Medicine, AMC Center for Heart Failure Research, Academic Medical Center, University of AmsterdamAmsterdam, The Netherlands; 2 Department of Anatomy, Embryology & Physiology, AMC Center for Heart Failure Research, Academic Medical Center, University of AmsterdamAmsterdam, The Netherlands; 3 Laboratory for Clinical Cardiovascular Physiology, AMC Center for Heart Failure Research, Academic Medical Center, University of AmsterdamAmsterdam, The Netherlands; 4 Department of Anesthesia, The Copenhagen Muscle Research Center, University of CopenhagenCopenhagen, Denmark; 5 Department of Endocrinology, Rigshospitalet, University of CopenhagenCopenhagen, Denmark; 6 MRC/Arthritis Research UK Centre for Musculoskeletal Ageing Research, School of Life Sciences, University of Nottingham Medical School, Queen's Medical CentreNottingham, UK

**Keywords:** Cardiac output, cerebral autoregulation, cerebral circulation, cerebral perfusion, cerebrovascular conductance

## Abstract

Endothelial vascular function and capacity to increase cardiac output during exercise are impaired in patients with type 2 diabetes (T2DM). We tested the hypothesis that the increase in cerebral blood flow (CBF) during exercise is also blunted and, therefore, that cerebral oxygenation becomes affected and perceived exertion increased in T2DM patients. We quantified cerebrovascular besides systemic hemodynamic responses to incremental ergometer cycling exercise in eight male T2DM and seven control subjects. CBF was assessed from the Fick equation and by transcranial Doppler-determined middle cerebral artery blood flow velocity. Cerebral oxygenation and metabolism were evaluated from the arterial-to-venous differences for oxygen, glucose, and lactate. Blood pressure was comparable during exercise between the two groups. However, the partial pressure of arterial carbon dioxide was lower at higher workloads in T2DM patients and their work capacity and increase in cardiac output were only ∽80% of that established in the control subjects. CBF and cerebral oxygenation were reduced during exercise in T2DM patients (*P* < 0.05), and they expressed a higher rating of perceived exertion (*P* < 0.05). In contrast, CBF increased ∽20% during exercise in the control group while the brain uptake of lactate and glucose was similar in the two groups. In conclusion, these results suggest that impaired CBF and oxygenation responses to exercise in T2DM patients may relate to limited ability to increase cardiac output and to reduced vasodilatory capacity and could contribute to their high perceived exertion.

## Introduction

Development of fatigue, defined as an exercise-induced loss of muscle force generating capacity, remains a problem in type 2 diabetes mellitus (T2DM) patients (Estacio et al. 1998; Taegtmeyer et al. 2002). Reduced exercise tolerance in T2DM is incompletely understood (Fang et al. 2005), and has been attributed to cardiac impairment (Poirier et al. 2000) and impaired muscle metabolism (Taegtmeyer et al. 2002; Scheuermann-Freestone et al. 2003). Rating of perceived exertion (RPE) increases with work rate as muscle oxygenation decreases (Mortensen et al. 2008), but a reduction in cerebral blood flow (CBF) and/or oxygenation could also be important determinants of RPE. Whether a reduction in cerebral perfusion and/or oxygenation limits exercise capacity is, however, not known (Fluck et al. 2014; Goodall et al. 2014).

CBF is critical for maintaining oxygen and substrate supply to the brain and is secured by several mechanisms, of which the partial pressure of arterial carbon dioxide (PaCO_2_), mean arterial pressure (MAP), and cerebral metabolism are the most important. When the brain is activated as during exercise, CBF and oxygenation increase (Ide et al. 1999b). An increase in near-infrared spectroscopy determined oxygenated hemoglobin (Hb) and a reduction in deoxygenated Hb in response to a motor task support that cerebral oxygenation exceeds the increase in O_2_ demand (Obrig et al. 1996). Comparable to the experience from functional MRI, cerebral activation elevates cerebral oxygenation (Hirth et al. 1997; Ide and Secher 2000). During exercise, the arterial O_2_ content may increase (Ide and Secher 2000) and, together with increased CBF in response to cerebral activation, enhance brain oxygen delivery (Jorgensen et al. 1992; Ide et al. 1999b; Secher et al. 2008). This increased oxygen delivery seems important since brain function deteriorates when its oxygenation is reduced which could play a role in the development of central fatigue with reduced motor drive to working muscles (Gonzalez-Alonso et al. 2004; Rasmussen et al. 2010). Cardiac output may also influence CBF during exercise (Ogoh et al. 2005; Secher et al. 2008). Inability to increase cardiac output sufficiently during exercise may jeopardize cerebral perfusion and thereby the ability of the central nervous system to drive the motoneurons adequately.

T2DM patients often have left ventricular diastolic dysfunction as a manifestation of diabetic cardiomyopathy (Brassard et al. 2007), and low stroke volume, hindering an adequate increase in cardiac output during exercise (Regensteiner et al. 2009). T2DM patients are also affected by impaired vasodilatory capacity for both systemic and cerebral vasculature, manifested by reduced cerebral CO_2_ responsiveness (Kim et al. 2011; Palazzo et al. 2013) and flow-mediated dilatation attributed to reduced nitric oxide bioavailability (Kingwell et al. 2003). Also, the cerebrovascular conductance response to exercise is attenuated in healthy subjects, when they are exposed to hyperglycemia (Kim et al. 2007). Therefore, there are several reasons why T2DM patients may be unable to increase CBF and cerebral oxygenation during exercise, which may set a limit to their exercise capacity.

To examine the hypothesis that the increase in CBF may be blunted in T2DM patients with a consequent decline in brain oxygenation, we compared CBF, cerebral oxygenation parameters and cerebral uptake of glucose and lactate from arterial-to-venous differences across the brain during incremental exercise in physically active male T2DM patients and in age- and sex-matched control subjects.

## Methods

### Subjects

Fully written informed consent was obtained from eight physically active T2DM male patients (61 ± 4 years, mean ± SD) and seven age- and sex-matched healthy subjects (56 ± 9 years; Table1) recruited by advertisement as approved by the local ethics committee (KF 01-090/01) in accordance with the Declaration of Helsinki. A physically active lifestyle was defined as self-reported participation of >2.5 h of (predominantly) cycling exercise per week, performed >10 years. T2DM patients were diagnosed according to WHO criteria and were treated with insulin and/or oral antidiabetic agents. Exclusion criteria were manifestations of cardiovascular disease comprising intracranial atherosclerosis, including transient ischemic attacks, stroke, heart failure, uncontrolled hypertension (blood pressure > 160/100 mm Hg), cardiovascular autonomic neuropathy, smoking, inadequate metabolic control (glycated hemoglobin >9.5%), and treatment with *β*-receptor adrenergic blocking agents (Ritz et al. 2014).

**Table 1:** Baseline characteristics of study population.

Characteristics	Control	Diabetes	*P*-value
Age (year)	56 ± 9	61 ± 4	0.28
Body mass index (kg·m^−2^)	27.5 ± 3.3	29.8 ± 5.8	0.36
Waist circumference (cm)	97 ± 13	105 ± 21	0.46
History of hypertension (*n*)	2	6	–
Systolic blood pressure (mm Hg)	133 ± 16	131 ± 16	0.75
Diastolic blood pressure (mm Hg)	72 ± 12	69 ± 11	0.57
Duration of diabetes (year)	–	8 ± 5	–
Microvascular complication			
Retinopathy	0	0	–
Nephropathy	0	0	–
Polyneuropathy (sensorimotor)	0	0	–
Oral hypoglycemic agents (metformin)	0	7 (7)	–
Insulin	0	2	–
Plasma glucose (mmol·L^−1^)	6.1 ± 0.1	7.5 ± 1.2**	<0.001
HbA_1c_ (% Hb)	5.4 ± 0.3	6.9 ± 0.9**	0.006
Antihypertensive drugs			
Angiotensin-converting enzyme inhibitor	1	3	–
Diuretic	1	4	–
Angiotensin II receptor antagonist	0	5	–
*β* blocker	0	0	–
Calcium channel blocker	0	3	–
Baseline hemodynamic parameters			
Mean arterial pressure (mm Hg)	79 ± 9	79 ± 13	1.00
Heart rate (bpm)	74 ± 12	76 ± 9	0.77
Stroke volume (mL)	79 ± 14	83 ± 24	0.68
Cardiac output (L·min^−1^)	5.7 ± 0.9	6.1 ± 2.3	0.81
Cardiac index (L·min^−1^·m^−2^)	2.69 ± 0.55	2.96 ± 1.05	0.71
Systemic vascular conductance (mL·min^−1^·mm Hg^−1^)	55 ± 8	61 ± 23	0.81
Systemic vascular conductance index (mL·min^−1^·mm Hg^−1^·m^−2^)	26 ± 5	30 ± 10	0.62
Cerebral vascular conductance (cm·sec^−1^·mm Hg^−1^)	0.53 ± 0.13	0.52 ± 0.12	0.91
Cerebral vascular conductance index (cm·sec^−1^·mm Hg^−1^·m^−2^)	0.25 ± 0.06	0.26 ± 0.08	0.84
MCA *V*_mean_ (cm·sec^−1^)	41 ± 7	41 ± 7	0.91
Cardiovascular autonomic function			
Forced respiratory sinus arrhythmia (I-E diff; bpm)	13 ± 7	17 ± 4	0.24
Normal blood pressure response to standing	7/7	8/8	–

HbA_1c_, glycated hemoglobin

MCA *V*_mean_, middle cerebral artery mean blood flow velocity

I-E diff, inspiratory-expiratory heart rate difference in beats·min^−1^.

** *P* < 0.01 versus control. Data are mean ± SD for *n* = 7 (control) versus *n* = 8 (diabetes).

### Experimental design

The subjects were requested to abstain from caffeinated beverages, alcohol, and heavy exercise for 12 h prior to reporting to the laboratory (room temperature ∽22°C) at 8:00 am after a light breakfast. In a slightly head-down position and under local anesthesia (lidocaine, 2%), a catheter (1.6 mm; 14 gauge; ES-04706, Arrow International, PA) was inserted retrograde in the right internal jugular vein guided by ultrasound and the catheter tip was advanced to the bulb of the vein. From that position blood was considered as being drained from the brain with, potentially, a small contribution from cerebrospinal fluid drained through the sagittal sinus. A second catheter (1.1 mm; 20 gauge) was inserted in the brachial artery of the nondominant arm. The catheter lumens were flushed (3 mL·h^−1^ isotonic saline) and arterial pressure was measured with a transducer (Edwards Lifesciences, Irvine, CA) zeroed at the level of the right atrium in the midaxillary line and connected to a pressure monitoring system (Dialogue 2000, Copenhagen, Denmark). After catheterization, the subjects rested for 1 h to offset influence of nociceptive stimuli on cerebral metabolism. The subjects then carried out incremental ergometer cycling exercise (Ergomedic 874E; Monark, Stockholm, Sweden; exercise capacity in Watts [W]) with each workload lasting 5 min separated by 5 min of recovery. Exercise started at 60 W and was increased by 30 W until the subjects were unable to maintain a pedaling rate of 60 rounds per minute despite verbal encouragement.

### Systemic and cerebral hemodynamics

Left ventricular stroke volume was assessed from the intra-arterial blood pressure waveform (BeatScope 1.0 software; BMEye, Amsterdam, The Netherlands) (Jellema et al. 1999). Cardiac output was stroke volume times heart rate, cardiac index was the ratio between cardiac output and body surface area, and systemic vascular conductance index was the ratio between cardiac output and MAP adjusted to body surface area.

Traditional global CBF measurement involves gas clearance techniques not suitable for exercise studies because of a low temporal resolution. In reverse, global CBF (CBF_FICK_ with oxygen as indicator) and cerebral metabolic rate of oxygen (CMRO_2_) are interdepending variables and two different estimates of CBF were made to account for this. First, changes in CBF were derived from the Fick principle: 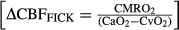 with CMRO_2_ set as 100% throughout the study and the arterio-jugular venous O_2_ difference determined from arterial and jugular venous samples simultaneously obtained at rest and in the last min of each workload and analyzed immediately (ABL 725, Radiometer, Copenhagen, Denmark) (Madsen et al. 1993; Rasmussen et al. 2010). Secondly, changes in the transcranial Doppler ultrasound determined middle cerebral artery (MCA) mean blood flow velocity (*V*_mean_) reflect CBF during exercise (CBF_TCD_) (Hellstrõm et al. 1996; Secher et al. 2008), assuming a constant vessel diameter for the range of changes in PaCO_2_ (Verbree et al. 2014) with CBF_TCD_ expressed relative to resting CBF set at 46 mL·100 g^−1^·min^−1^ (Madsen et al. 1993; Rasmussen et al. 2010). CMRO_2_ was then determined from the arterio-venous difference for O_2_, multiplied by CBF_TCD_, adjusted in proportion to changes in MCA *V*_mean_: 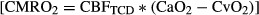. The cerebrovascular conductance index was the ratio between MCA *V*_mean_ and MAP (Immink et al. 2004) adjusted to body surface area and cerebral O_2_ extraction ratio was (CaO_2_-CvO_2_)/CaO_2_.

### Cerebral oxygenation variables

Changes in cerebral oxygenation were expressed as brain capillary O_2_ saturation (*S*_cap_O_2_), brain capillary O_2_ tension (*P*_cap_O_2_), and brain mitochondrial O_2_ tension (*P*_Mito_O_2_) (Gjedde et al. 2005; Rasmussen et al. 2007, 2012). The *S*_cap_O_2_ was calculated as: 
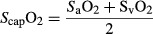


where *S*_a_O_2_ is the arterial O_2_ saturation, and *S*_v_O_2_ the internal jugular venous O_2_ saturation (Gjedde et al. 2005; Rasmussen et al. 2007, 2010).

With the assumption that capillary recruitment does not manifest within the brain, *P*_cap_O_2_ is 
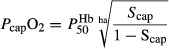


where 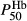 is the capillary Po_2_ when hemoglobin is half saturated and h_a_ is the Hill coefficient for arterial blood. The 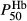 was estimated as the average of arterial and venous *P*_50_ (ABL 725 Radiometer) and *h*_a_ was calculated as 
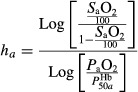


The *P*_Mito_O_2_ depends on the balance between the brain's O_2_ supply, O_2_ extraction, and O_2_ conductance from the capillary to the mitochondria (*L*). *P*_Mito_O_2_ is determined from *P*_cap_O_2_, CMRO_2_, and oxygen diffusability (*L*) as 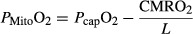


### Cerebrovascular autoregulation and cardiovascular autonomic function

Prior to cycling exercise the subjects underwent evaluation of dynamic cerebrovascular autoregulation and cardiovascular autonomic function. Dynamic cerebrovascular autoregulation was quantified as the counterregulatory capacity to maintain MCA *V*_mean_ during spontaneous blood pressure oscillations. A 5-min tracing of beat-to-beat data of MAP and MCA *V*_mean_ was spline interpolated and resampled at 4 Hz. Variability in MAP and MCA *V*_mean_ was estimated with discrete Fourier transformation and from the cross spectrum the phase shift of the MAP to MCA *V*_mean_ transfer function and its gain were derived. The gain was the ratio of the amplitudes of MCA *V*_mean_ and MAP, reflecting the effective dampening expressed as change (cm·sec^−1^) per change in mm Hg in the low frequency range (0.07 to 0.15 Hz) (Immink et al. 2004). Coherence examined the strength of the relationship between MAP and MCA *V*_mean_; only data with coherence >0.5 were included in the analysis (Immink et al. 2004). Parasympathetic control of the heart was quantified by forced respiratory sinus arrhythmia and the heart rate response to standing up, while sympathetic cardiovascular control was assessed by monitoring the postural blood pressure response (Wieling and Van Lieshout 1997).

### Data analysis

Signals were analog-to-digital-converted at 200 Hz and stored on hard disk for off-line analysis with beat-to-beat values for MCA *V*_mean_ and MAP derived as the integral over one beat divided by the corresponding beat interval. Baseline hemodynamic parameters were derived in the sitting resting position. Values were expressed as 30-sec averages over the last min at each workload when the subjects reported their RPE (Borg scale) (Borg 1970). Data are presented as mean (±SD) and differences between groups were identified by unpaired Student *t*-test when data fitted a normal distribution, while a Mann–Whitney rank sum test was applied when data were not normally distributed. Differences between groups (diabetes vs. control) and workloads were evaluated by two-way repeated measures ANOVA. A *post hoc* Bonferroni analysis was applied for multiple comparisons, when a statistically significant deviation (*P* < 0.05) was detected.

## Results

Subject characteristics were comparable for T2DM patients and control subjects except for plasma glucose (*P* < 0.01) and glycated hemoglobin (*P* < 0.01) (Table1). Resting cerebrovascular and systemic cardiovascular variables did not differ between groups. For none of the subjects more than two abnormal autonomic cardiovascular test results were identified and the subjects were thereby not considered to suffer from cardiovascular autonomic neuropathy. Variability in MAP and MCA *V*_mean_, and phase and gain of the MAP–MCA *V*_mean_ transfer function did not significantly differ between groups (Table2). Also, resting arterial blood gas variables, net brain arterio-venous differences in O_2_, glucose and lactate, as well as O_2_ extraction ratio and CMRO_2_ were comparable between patients and controls (Table3).

**Table 2:** Transfer function gain, phase, and coherence.

Low frequency (0.07–0.15 Hz)	CTRL (*n* = 6)	T2DM (*n* = 6)	*P*-value
MAP power, mm Hg^2^·Hz^−1^	12 ± 8	8 ± 10	0.240
MCA *V*_mean_ power, (cm·sec^−1^)^2^·Hz^−1^	3.9 ± 3.4	2.3 ± 3.1	0.180
Coherence, k^2^	0.85 ± 0.06	0.77 ± 0.13	0.230
Phase, degrees	33 ± 13	23 ± 9	0.137
Gain, cm·sec^−1^·mm Hg^−1^	0.54 ± 0.10	0.47 ± 0.05	0.217

Data are presented as mean ± SD.

**Table 3:** Arterial blood gas, metabolic variables, and brain oxygenation at rest and during exercise.

	Group	Rest	Ex 60	Ex 90	Ex 120	Ex 150	Max
*Arterial blood*							
*p*H	Control	7.42 ± 0.03	7.40 ± 0.03	7.40 ± 0.03	7.39 ± 0.04†	7.37 ± 0.05†	7.35 ± 0.06†
	Diabetes	7.42 ± 0.02	7.40 ± 0.03†	7.40 ± 0.02†	7.39 ± 0.03‡	7.38 ± 0.03‡	7.36 ± 0.04‡
PaO_2_ (kPa)	Control	13.0 ± 1.0	13.2 ± 0.8	13.1 ± 0.7	12.5 ± 0.6	12.6 ± 0.9	13.0 ± 0.9
	Diabetes	12.1 ± 1.1	12.7 ± 1.2	13.0 ± 1.0†	13.1 ± 1.5†	13.2 ± 1.2‡	13.0 ± 1.8
PaCO_2_ (kPa)	Control	5.1 ± 0.4	5.3 ± 0.4	5.2 ± 0.4	5.0 ± 0.4	4.7 ± 0.4	4.0 ± 0.3‡
	Diabetes	5.1 ± 0.4	5.2 ± 0.5	5.0 ± 0.5	4.6 ± 0.6‡	4.2 ± 0.7‡	3.9 ± 0.5‡
SO_2_ (%)	Control	97.9 ± 0.5	97.9 ± 0.7	97.8 ± 0.6	97.5 ± 0.4	97.5 ± 0.5	97.4 ± 0.9
	Diabetes	97.5 ± 0.7	97.8 ± 0.7	97.9 ± 0.7	98.0 ± 0.8	97.8 ± 0.8	97.5 ± 1.4
CaO_2_ (mL·L^−1^)	Control	201 ± 19	206 ± 18†	207 ± 19†	208 ± 19†	209 ± 18†	213 ± 20‡
	Diabetes	208 ± 12	211 ± 13†	212 ± 13	214 ± 14†	216 ± 15†	218 ± 16‡
Glucose (mmol·L^−1^)	Control	6.1 ± 0.1	6.0 ± 0.2	6.2 ± 0.3	6.0 ± 0.5	5.9 ± 0.4	6.2 ± 0.5
	Diabetes	7.5 ± 1.2**	7.6 ± 1.3**	7.6 ± 1.6*	7.6 ± 1.7*	7.7 ± 2.0*	7.8 ± 1.9*
*Brain A-V difference*							
O_2_ (mmol·L^−1^)	Control	2.8 ± 0.7	2.5 ± 0.6	2.5 ± 0.7	2.6 ± 1.0	2.9 ± 0.9	3.5 ± 0.6†
	Diabetes	3.2 ± 0.7	3.0 ± 0.9	3.4 ± 1.0	3.8 ± 1.2†	4.4 ± 1.3‡,*	4.8 ± 1.1‡,*
Glucose (mmol·L^−1^)	Control	0.5 ± 0.2	0.4 ± 0.2	0.6 ± 0.2	0.6 ± 0.2	0.6 ± 0.2	0.7 ± 0.2†
	Diabetes	0.6 ± 0.2	0.6 ± 0.2*	0.7 ± 0.1	0.8 ± 0.2†	0.8 ± 0.1†,*	1.0 ± 0.8
Lactate (mmol·L^−1^)	Control	0.0 ± 0.2	0.2 ± 0.1	0.2 ± 0.1	0.4 ± 0.2‡	0.6 ± 0.2‡	1.0 ± 0.5†
	Diabetes	0.0 ± 0.1	0.1 ± 0.2	0.1 ± 0.4	0.5 ± 0.6‡	0.6 ± 0.5‡	1.4 ± 1.9†
O_2_ extraction ratio	Control	0.31 ± 0.10	0.29 ± 0.12	0.29 ± 0.13	0.31 ± 0.15	0.33 ± 0.15	0.42 ± 0.10
	Diabetes	0.35 ± 0.07	0.34 ± 0.06	0.36 ± 0.10	0.40 ± 0.12	0.47 ± 0.10‡^,^*	0.49 ± 0.10‡
CMRO_2_ (μmol·100 g^−1^·min^−1^)	Control	149 ± 42	142 ± 35	146 ± 47	161 ± 62	168 ± 59	191 ± 56†
	Diabetes	150 ± 30	142 ± 47	154 ± 46	164 ± 52	180 ± 64	191 ± 75

PaO_2_, arterial oxygen tension

PaCO_2_, arterial carbon dioxide tension

SO_2_, hemoglobin oxygen saturation

CaO_2_, arterial oxygen content

A-V difference, arterio-jugular venous difference

CMRO_2_, cerebral metabolic rate for oxygen.

† *P* < 0.05 versus rest

‡ *P* < 0.01 versus rest

* *P* < 0.05 versus control

** *P* < 0.01 versus control. Values are mean ± SD for *n* = 7 (control) versus *n* = 8 (diabetes).

### Hemodynamic responses and work capacity

**Figure 1:**
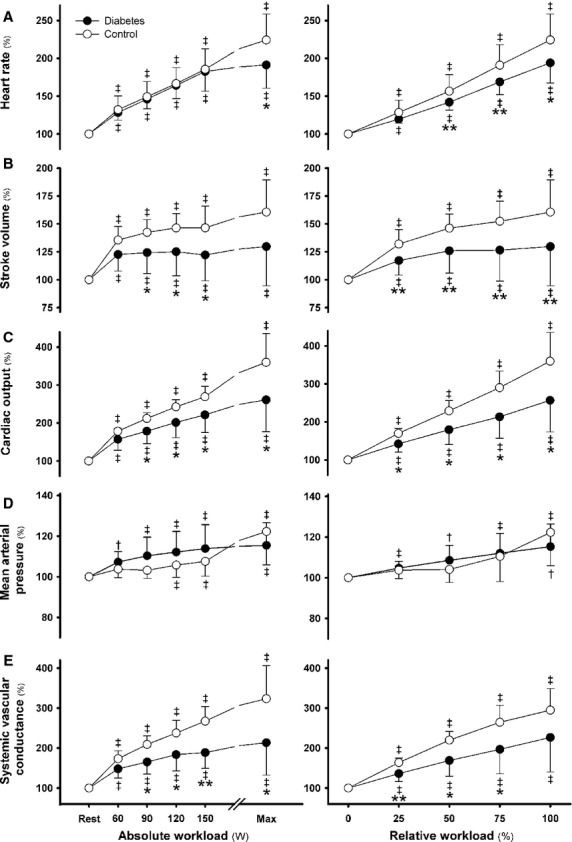
Cardiovascular hemodynamics. Heart rate (A), Stroke volume (B), Cardiac output (C), Mean arterial pressure (D), Systemic vascular conductance (E) from rest to maximal exercise in type 2 diabetic patients (closed circles) versus control subjects (open circles). Left panels: Absolute workload; Right panels: Relative workload. ^†^*P* < 0.05 and ^‡^*P* < 0.01 versus rest; *P < 0.05 and **P < 0.01 versus control subjects. Values are mean ± SD.

**Figure 2:**
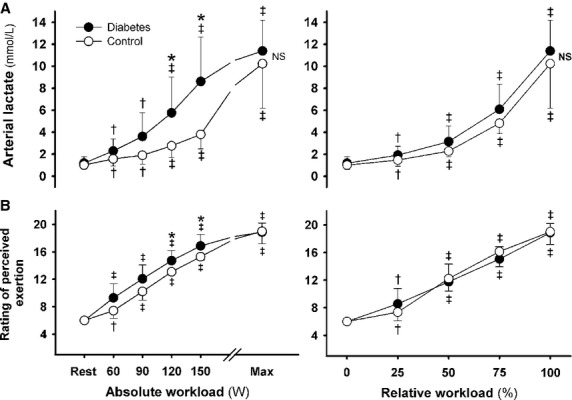
Arterial lactate concentration and rating of perceived exertion. Arterial lactate concentration (A) and rating of perceived exertion (B). Type 2 diabetic patients (closed circles) versus control subjects (open circles) at the same absolute (left panels) and relative workload (right panels). ^†^*P* < 0.05 and ^‡^*P* < 0.01 versus rest; *P < 0.05 versus control subjects. Values are mean ± SD.

At each workload, exercise induced a comparable increase in MAP in the two groups, but the increase in heart rate for relative versus absolute workloads was smaller in T2DM patients versus controls (*P* < 0.05; Fig.1). Together with a smaller stroke volume, the increase in cardiac output was attenuated by 20% and matched by a 20% lower maximal workload (169 ± 32 vs. 214 ± 36 W; *P* < 0.05). The increase in systemic vascular conductance was smaller in the T2DM group for both absolute and relative workloads. The RPE was higher in T2DM versus controls for 120 and 150 W (*P* < 0.05; Fig.2B). Of note, arterial lactate concentration was higher for each absolute workload but comparable for relative and maximal workload (Fig.2A).

### Cerebral perfusion and oxygenation

**Figure 3:**
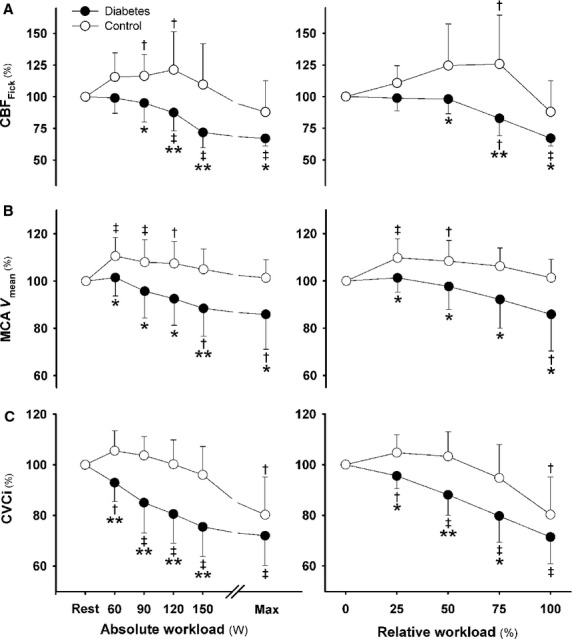
Cerebrovascular hemodynamics. Cerebral blood flow derived from the Fick principle from inverse arterial-jugular venous oxygen difference (A), middle cerebral artery mean blood flow velocity (B), cerebrovascular conductance index (C). Type 2 diabetic patients (closed circles) versus control subjects (open circles) at the same absolute (left panels) and relative workload (right panels). ^†^*P* < 0.05 and ^‡^*P* < 0.01 versus rest; *P < 0.05 and **P < 0.01 versus control subjects. Values are mean ± SD.

**Figure 4:**
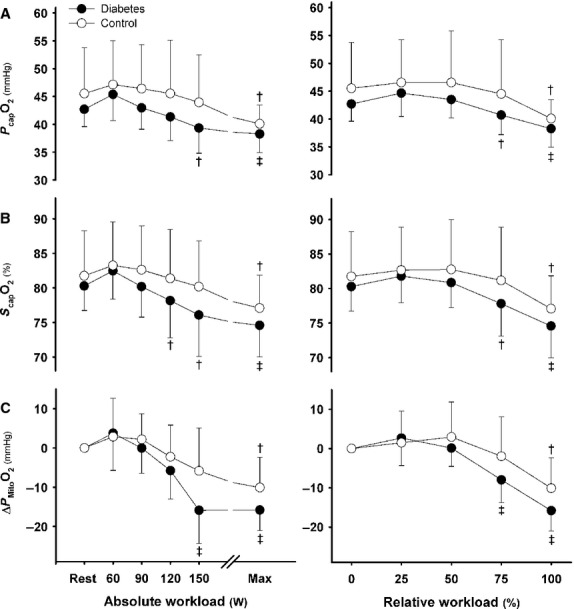
Brain oxygenation. Brain capillary oxygen tension (A), brain capillary oxygen saturation (B), cerebral mitochondrial oxygen tension (C). Type 2 diabetic patients (closed circles) versus control subjects (open circles) at the same absolute (left panels) and relative workload (right panels). ^†^*P* < 0.05 and ^‡^*P* < 0.01 versus rest. Values are mean ± SD.

CBF_Fick_ and MCA *V*_mean_ increased with exercise intensity in the control group (Fig.3), but declined early in T2DM with a reduction in cerebrovascular conductance index (*P* < 0.01 vs. controls). From 150 W on, cerebral O_2_ extraction was higher in T2DM (*P* < 0.05 vs. controls; Table3). CMRO_2_ increased ∽30% at the highest work intensities in both groups of subjects, which together with the lower cerebral perfusion in T2DM patients, decreased *S*_cap_O_2_, *P*_cap_O_2,_ and *P*_Mito_O_2_ (*P* < 0.05; Fig.4).

### Cerebral substrate use

**Figure 5:**
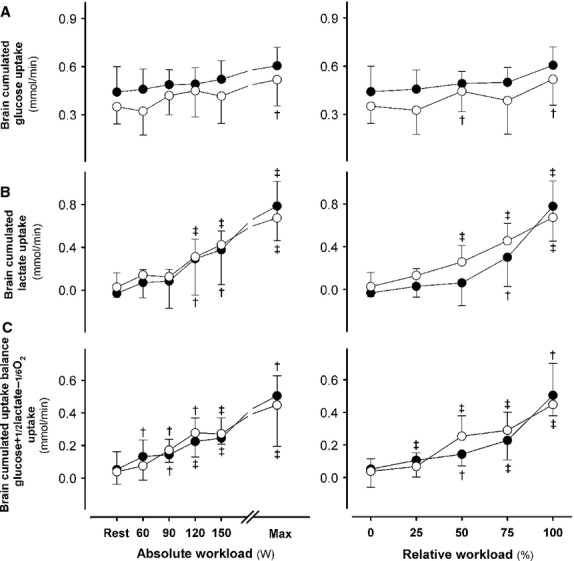
Brain metabolic response. Brain cumulated uptake rates of glucose (A) and lactate (B), and the total carbohydrate uptake balance (C). Type 2 diabetic patients (closed circles) versus control subjects (open circles) at the same absolute (left panels) and relative workload (right panels). ^†^*P* < 0.05 and ^‡^*P* < 0.01 versus rest. Values are mean ± SD.

During intense exercise the increase in the arterio-venous lactate difference across the brain, the cumulated cerebral glucose and lactate uptake rate, and the calculated brain carbohydrate to O_2_ uptake balances were comparable between the two groups (Fig.5).

## Discussion

The novel findings of this study were threefold. Firstly, male patients with T2DM without symptomatic cardiovascular disease exhibited reduced cerebral perfusion and oxygenation during incremental exercise associated with attenuated increases in cerebral and systemic vascular conductance compared with nondiabetic-matched controls. Secondly, cerebral oxygenation reached its lowest level at exhaustion at a 20% lower workload in T2DM patients than healthy controls and T2DM patients expressed a higher RPE than healthy controls. Finally, CMRO_2_ and cerebral uptake of carbohydrate were maintained in T2DM patients despite the blunted increase in cerebral perfusion during exercise. Accordingly, early reduction in cerebral capillary oxygenation and *P*_Mito_O_2,_ rather than deranged brain metabolism could represent a limiting factor for exercise capacity and influence RPE. Together, these findings provide an incremental step forward in our understanding of the impact of T2DM on cardio- and cerebrovascular physiology during exercise.

Normative aging is associated with reductions in global and regional CBF and in cerebral metabolism affecting gray matter flow with a ∽15% reduction between the 3rd and 5th decade (Shaw et al. 1984). The systemic (Phillips et al. 2012) and cerebral (Fisher et al. 2013) vascular conductance responses to exercise are mitigated with aging and the results of the present study suggest that the increase in CBF during exercise is even lower in T2DM patients. That was the case although T2DM patients demonstrated no signs of cardiovascular autonomic neuropathy and their dynamic cerebrovascular autoregulatory capacity was not affected.

Healthy subjects demonstrated an increase in cerebral perfusion at low workloads followed by a decline in cerebral perfusion and oxygenation with hyperventilation-induced hypocapnia and subsequent vasoconstriction at higher exercise intensities. In contrast, a reduction in brain perfusion was observed even at low levels of exercise in T2DM patients, at a comparable PaCO_2_ in the two groups.

In healthy subjects, hyperglycemia is associated with reduced glycocalyx volume and impaired endothelium-dependent flow-mediated dilatation due to reduced nitric oxide availability and cerebrovascular conductance is attenuated during exercise (Kim et al. 2007). T2DM patients demonstrate impaired endothelium-dependent vasodilatation with attenuated increase in limb blood flow during exercise (Kingwell et al. 2003). Furthermore, cerebral vasodilation is impaired in T2DM, reflected by reduced CO_2_ responsiveness, and associated with endothelial dysfunction, even without overt microvascular complications (Lavi et al. 2006; Kim et al. 2011), which also may have contributed to the blunted increase in CBF during exercise.

Cardiac output is important for cerebral perfusion during exercise, independent of arterial pressure (Ide et al. 1998; Van Lieshout et al. 2001; Ogoh et al. 2005; Secher et al. 2008). For example, the increase in cardiac output during exercise is attenuated following administration of a *β*-adrenergic blocking agent and the increase in cerebral perfusion is reduced to about half of the normal response (Ide et al. 1998). Similarly, in patients with cardiac insufficiency, there is a relationship between the ability to increase cardiac output and cerebral perfusion during exercise (Ide et al. 1999a). CBF is also reversibly reduced in patients with severe heart failure, substantiated by a significant increase in CBF after heart transplantation (Gruhn et al. 2001). We consider that the attenuated increase in cardiac output in T2DM patients in response to exercise may be a consequence of their cardiac dysfunction (Regensteiner et al. 2009) and potentially a result of impaired myocardial muscle energy metabolism (Scheuermann-Freestone et al. 2003), as well as attenuated increase in skeletal muscle blood flow (Bada et al. 2012). Tight coupling between cardiac output and O_2_ uptake is supported by the 20% lower cardiac output and work capacity in T2DM patients and higher blood lactate levels at any given absolute workload. This study conforms to data demonstrating a reduced capacity to increase cardiac performance during exercise in T2DM patients (Pinto et al. 2014). Due to a limited potential for capillary recruitment within the brain (Ide et al. 1999b), its O_2_ supply depends on the capacity to increase CBF. Accordingly, limited exercise capacity in T2DM patients may be attributed to their inability to increase cerebrovascular vascular conductance, which in turn could be a consequence of their limitation in cardiac performance.

*P*_mito_O_2_ is a global estimate of cerebral oxygenation and a reduction by more than ∽5 mm Hg is associated with elevated cerebral lactate production and reduced work capacity (Rasmussen et al. 2007). *P*_Mito_O_2_ declines during intense exercise, which is the consequence of a reduction in cerebral perfusion and an increase in CMRO_2_ (Secher et al. 2008). A mismatch between neuronal activity and O_2_ delivery during exercise and the accompanying fall in cerebral oxygenation reflected in *P*_Mito_O_2_ may affect activation of exercising muscles, that is, induce so-called central fatigue (Rasmussen et al. 2007, 2010; Secher et al. 2008). Exhaustive exercise provokes cerebral deoxygenation and indices of supraspinal fatigue similar to what is observed during exercise in hypoxia (Imray et al. 2005; Nybo and Rasmussen 2007; Goodall et al. 2014), indicating that reduced cerebral oxygenation is important for development of fatigue (Rasmussen et al. 2007, 2010). For the T2DM patients included in this study, cerebral oxygenation declined together with cerebral perfusion in the early stages of incremental exercise. The finding that cerebral oxygenation reached its lowest level at exhaustion supports that cerebral oxygenation is a critical factor for dynamic exercise (Secher et al. 2008; Rasmussen et al. 2010). However, the extent to which the low level of cerebral oxygenation at exhaustion provokes fatigue remains debated (Rupp and Perrey 2008; Subudhi et al. 2011).

Coupling between cerebral perfusion and metabolic demand becomes altered during somatosensory stimulation (Fox and Raichle 1986). The brain uptake rate for glucose and lactate was comparable among groups, indicating that the blunted CBF response in T2DM patients did not affect the brain's nonoxidative carbohydrate consumption. No single cause of fatigue has been identified and fatigue is, therefore, considered to be multifactorial with focus on skeletal muscle abnormalities. For instance T2DM skeletal muscles demonstrate a transient imbalance of muscle O_2_ delivery relative to O_2_ uptake after onset of exercise attributed to impaired vasodilatation (Bauer et al. 2007). The increase in plasma lactate during exercise for a given absolute workload was larger in patients with T2DM that, together with their high RPE, conform to a low work capacity. Yet, blood lactate may reflect the hepato-splanchnic lactate elimination rate affected by metformin in T2DM patients.

## Limitations

Consideration must be given to the variable effects of antihypertensive agents on CBF. The cerebral circulation has angiotensin receptors that may account for the improved CBF and favorable autoregulatory responses in hypertensive patients treated with angiotensin-converting enzyme inhibitors and angiotensin receptor blockers. Also, calcium channel blockers increase CBF and are used to treat cerebral vasospasms after subarachnoid hemorrhage. Beta blockade blunts the cardiac output response to exercise (Ide et al. 1998), and their use was an exclusion criterion for this study. Thus, any effect of antihypertensive agents during exercise should lead to augmenting CBF and although more T2DM patients were on antihypertensive medication, their increase in CBF during exercise was blunted. Hypertension and diabetes both contribute to remodeling of systemic resistance arteries with a reduced peripheral vascular conductance (Schofield et al. 2002). Although hypertension treatment (angiotensin-converting enzyme inhibitors, angiotensin receptor blockers, and calcium channel blockers) may have ameliorable vascular effects in the T2DM patients, their leg vascular conductance response to exercise-induced hyperemia was lower compared to the healthy subjects. We therefore consider it unlikely that attenuated cardiac output and CBF in the T2DM patients are due to medication, and the T2DM patients were selected among the healthiest segment of that population, that is, without clinical signs of micro- or macrovascular complications.

We studied male subjects to eliminate sex-related variation given that the number of subjects included in invasive studies is limited. Applicability of the data to female patients with T2DM remains therefore uncertain, but blood flow responses to hyperemia in women with T2DM are reported to be lower than in men (Regensteiner et al. 2015). We consider that the discrepancy between the high-normal baseline arterial glucose concentration and the normal HbA_1c_ level in the control group suggests a stress-mediated metabolic response rather than impaired fasting glucose. Given the inherent limitations associated with employing brain-imaging techniques during vigorous dynamic exercise, we used arterial-to-internal jugular venous differences to assess cerebral perfusion and oxygenation. CMRO_2_ is considered to remain unchanged during ergometer cycling for as long as the intensity is moderate, whereas during intense exercise CMRO_2_ may increase, as found in this study, with some underestimate of global CBF (Thomas et al. 1989; Jorgensen et al. 1992; Ide et al. 1999b; Gonzalez-Alonso et al. 2004; Seifert et al. 2008; Laughlin et al. 2012). We consider that with a similar increase in CMRO_2_ in both groups the observed difference in CBF response is still valid.

The subjects were not screened for coronary artery disease but performed maximal exercise without symptoms of cardiac ischemia. During incremental exercise RPE was higher in T2DM patients who were ∽5 years older with a slightly higher body mass index, but there was no relation between the cerebrovascular response and age or body mass index. Importantly, the differences in hemodynamic responses reported in the present study were quantitatively large (∽20%) between groups, despite the small sample size due to the invasive nature of this study.

## Conclusions

These results suggest that impaired CBF and oxygenation responses to exercise may relate to a limited ability to increase cardiac output and to reduced vasodilatory capacity and could contribute to increased perceived exertion in T2DM patients.

## References

[b1] Bada AA, Svendsen JH, Secher NH, Saltin B, Mortensen SP (2012). Peripheral vasodilation determines cardiac output in exercising humans: insight from atrial pacing. J. Physiol.

[b2] Bauer TA, Reusch JE, Levi M, Regensteiner JG (2007). Skeletal muscle deoxygenation after the onset of moderate exercise suggests slowed microvascular blood flow kinetics in type 2 diabetes. Diabetes Care.

[b3] Borg G (1970). Perceived exertion as an indicator of somatic stress. Scand. J. Rehabil. Med.

[b4] Brassard P, Legault S, Garneau C, Bogaty P, Dumesnil JG, Poirier P (2007). Normalization of diastolic dysfunction in type 2 diabetics after exercise training. Med. Sci. Sports Exerc.

[b5] Estacio RO, Regensteiner JG, Wolfel EE, Jeffers B, Dickenson M, Schrier RW (1998). The association between diabetic complications and exercise capacity in NIDDM patients. Diabetes Care.

[b6] Fang ZY, Sharman J, Prins JB, Marwick TH (2005). Determinants of exercise capacity in patients with type 2 diabetes. Diabetes Care.

[b7] Fisher JP, Hartwich D, Seifert T, Olesen ND, McNulty CL, Nielsen HB (2013). Cerebral perfusion, oxygenation and metabolism during exercise in young and elderly individuals. J. Physiol.

[b8] Fluck D, Braz ID, Keiser S, Huppin F, Haider T, Hilty M (2014). Age, aerobic fitness and cerebral perfusion during exercise: role of carbon dioxide. Am. J. Physiol. Heart Circ. Physiol.

[b9] Fox PT, Raichle ME (1986). Focal physiological uncoupling of cerebral blood flow and oxidative metabolism during somatosensory stimulation in human subjects. Proc. Natl Acad. Sci. USA.

[b10] Gjedde A, Johannsen P, Cold GE, Ostergaard L (2005). Cerebral metabolic response to low blood flow: possible role of cytochrome oxidase inhibition. J. Cereb. Blood Flow Metab.

[b11] Gonzalez-Alonso J, Dalsgaard MK, Osada T, Volianitis S, Dawson EA, Yoshiga CC (2004). Brain and central hemodynamics and oxygenation during maximal exercise in humans. J. Physiol.

[b12] Goodall S, Twomey R, Amann M, Ross EZ, Lovering AT, Romer LM (2014). AltitudeOmics: exercise-induced supraspinal fatigue is attenuated in healthy humans after acclimatization to high altitude. Acta Physiol.

[b13] Gruhn N, Larsen FS, Boesgaard S, Knudsen GM, Mortensen SA, Thomsen G (2001). Cerebral blood flow in patients with chronic heart failure before and after heart transplantation. Stroke.

[b14] Hellstrõm G, Fischer-Colbrie W, Wahlgren NG, Jøgestrand T (1996). Carotid artery blood flow and middle cerebral artery blood flow velocity during physical exercise. J. Appl. Physiol.

[b15] Hirth C, Obrig H, Valdueza J, Dirnagl U, Villringer A (1997). Simultaneous assessment of cerebral oxygenation and hemodynamics during a motor task. A combined near infrared and transcranial Doppler sonography study. Adv. Exp. Med. Biol.

[b16] Ide K, Secher NH (2000). Cerebral blood flow and metabolism during exercise. Prog. Neurobiol.

[b17] Ide K, Pott F, Van Lieshout JJ, Secher NH (1998). Middle cerebral artery blood velocity depends on cardiac output during exercise with a large muscle mass. Acta Physiol. Scand.

[b18] Ide K, Gullov AL, Pott F, Van Lieshout JJ, Koefoed BG, Petersen P (1999a). Middle cerebral artery blood velocity during exercise in patients with atrial fibrillation. Clin. Physiol.

[b19] Ide K, Horn A, Secher NH (1999b). Cerebral metabolic response to submaximal exercise. J. Appl. Physiol.

[b20] Immink RV, van den Born BJ, van Montfrans GA, Koopmans RP, Karemaker JM, van Lieshout JJ (2004). Impaired cerebral autoregulation in patients with malignant hypertension. Circulation.

[b21] Imray CH, Myers SD, Pattinson KT, Bradwell AR, Chan CW, Harris S (2005). Effect of exercise on cerebral perfusion in humans at high altitude. J. Appl. Physiol.

[b22] Jellema WT, Wesseling KH, Groeneveld AB, Stoutenbeek CP, Thijs LG, van Lieshout JJ (1999). Continuous cardiac output in septic shock by simulating a model of the aortic input impedance: a comparison with bolus injection thermodilution. Anesthesiology.

[b23] Jorgensen LG, Perko G, Secher NH (1992). Regional cerebral artery mean flow velocity and blood flow during dynamic exercise in humans. J. Appl. Physiol.

[b24] Kim YS, Krogh-Madsen R, Rasmussen P, Plomgaard P, Ogoh S, Secher NH (2007). Effects of hyperglycemia on the cerebrovascular response to rhythmic handgrip exercise. Am. J. Physiol. Heart Circ. Physiol.

[b25] Kim YS, Davis SC, Truijen J, Stok WJ, Secher NH, van Lieshout JJ (2011). Intensive blood pressure control affects cerebral blood flow in type 2 diabetes mellitus patients. Hypertension.

[b26] Kingwell BA, Formosa M, Muhlmann M, Bradley SJ, McConell GK (2003). Type 2 diabetic individuals have impaired leg blood flow responses to exercise - Role of endothelium-dependent vasodilation. Diabetes Care.

[b27] Laughlin MH, Davis MJ, Secher NH, van Lieshout JJ, Arce-Esquivel AA, Simmons GH (2012). Peripheral circulation. Compr Physiol.

[b28] Lavi S, Gaitini D, Milloul V, Jacob G (2006). Impaired cerebral CO2 vasoreactivity: association with endothelial dysfunction. Am. J. Physiol. Heart Circ. Physiol.

[b29] Madsen PL, Holm S, Herning M, Lassen NA (1993). Average blood flow and oxygen uptake in the human brain during resting wakefulness: a critical appraisal of the Kety-Schmidt technique. J. Cereb. Blood Flow Metab.

[b30] Mortensen SP, Damsgaard R, Dawson EA, Secher NH, Gonzalez-Alonso J (2008). Restrictions in systemic and locomotor skeletal muscle perfusion, oxygen supply and VO2 during high-intensity whole-body exercise in humans. J. Physiol.

[b31] Nybo L, Rasmussen P (2007). Inadequate cerebral oxygen delivery and central fatigue during strenuous exercise. Exerc. Sport Sci. Rev.

[b32] Obrig H, Hirth C, Junge-Hulsing JG, Doge C, Wolf T, Dirnagl U (1996). Cerebral oxygenation changes in response to motor stimulation. J. Appl. Physiol.

[b33] Ogoh S, Brothers RM, Barnes Q, Eubank WL, Hawkins MN, Purkayastha S (2005). The effect of changes in cardiac output on middle cerebral artery mean blood velocity at rest and during exercise. J. Physiol.

[b34] Palazzo P, Maggio P, Altavilla R, Di Flaviani A, Giordani I, Malandrucco I (2013). Cerebral hemodynamics and systemic endothelial function are already impaired in well-controlled type 2 diabetic patients, with short-term disease. PLoS ONE.

[b35] Phillips B, Williams J, Atherton P, Smith K, Hildebrandt W, Rankin D (2012). Resistance exercise training improves age-related declines in leg vascular conductance and rejuvenates acute leg blood flow responses to feeding and exercise. J. Appl. Physiol.

[b36] Pinto TE, Gusso S, Hofman PL, Derraik JG, Hornung TS, Cutfield WS (2014). Systolic and diastolic abnormalities reduce the cardiac response to exercise in adolescents with type 2 diabetes. Diabetes Care.

[b37] Poirier P, Garneau C, Bogaty P, Nadeau A, Marois L, Brochu C (2000). Impact of left ventricular diastolic dysfunction on maximal treadmill performance in normotensive subjects with well-controlled type 2 diabetes mellitus. Am. J. Cardiol.

[b38] Rasmussen P, Dawson EA, Nybo L, van Lieshout JJ, Secher NH, Gjedde A (2007). Capillary-oxygenation-level-dependent near-infrared spectrometry in frontal lobe of humans. J. Cereb. Blood Flow Metab.

[b39] Rasmussen P, Nielsen J, Overgaard M, Krogh-Madsen R, Gjedde A, Secher NH (2010). Reduced muscle activation during exercise related to brain oxygenation and metabolism in humans. J. Physiol.

[b40] Rasmussen P, Kim YS, Krogh-Madsen R, Lundby C, Olsen NV, Secher NH (2012). Both acute and prolonged administration of EPO reduce cerebral and systemic vascular conductance in humans. FASEB J.

[b41] Regensteiner JG, Bauer TA, Reusch JE, Quaife RA, Chen MY, Smith SC (2009). Cardiac dysfunction during exercise in uncomplicated type 2 diabetes. Med. Sci. Sports Exerc.

[b42] Regensteiner JG, Bauer TA, Huebschmann AG, Herlache L, Weinberger HD, Wolfel EE (2015). Sex Differences in the Effects of Type 2 Diabetes on Exercise Performance. Med. Sci. Sports Exerc.

[b43] Ritz K, Denswil NP, Stam OC, van Lieshout JJ, Daemen MJ (2014). Cause and mechanisms of intracranial atherosclerosis. Circulation.

[b44] Rupp T, Perrey S (2008). Prefrontal cortex oxygenation and neuromuscular responses to exhaustive exercise. Eur. J. Appl. Physiol.

[b45] Scheuermann-Freestone M, Madsen PL, Manners D, Blamire AM, Buckingham RE, Styles P (2003). Abnormal cardiac and skeletal muscle energy metabolism in patients with type 2 diabetes. Circulation.

[b46] Schofield I, Malik R, Izzard A, Austin C, Heagerty A (2002). Vascular structural and functional changes in type 2 diabetes mellitus: evidence for the roles of abnormal myogenic responsiveness and dyslipidemia. Circulation.

[b47] Secher NH, Seifert T, Van Lieshout JJ (2008). Cerebral blood flow and metabolism during exercise: implications for fatigue. J. Appl. Physiol.

[b48] Seifert T, Rasmussen P, Secher NH, Nielsen HB (2008). Cerebral oxygenation decreases during exercise in humans with beta-adrenergic blockade. Acta Physiol.

[b49] Shaw TG, Mortel KF, Meyer JS, Rogers RL, Hardenberg J, Cutaia MM (1984). Cerebral blood flow changes in benign aging and cerebrovascular disease. Neurology.

[b50] Subudhi AW, Olin JT, Dimmen AC, Polaner DM, Kayser B, Roach RC (2011). Does cerebral oxygen delivery limit incremental exercise performance?. J. Appl. Physiol.

[b51] Taegtmeyer H, McNulty P, Young ME (2002). Adaptation and maladaptation of the heart in diabetes: Part I: general concepts. Circulation.

[b52] Thomas SN, Schroeder T, Secher NH, Mitchell JH (1989). Cerebral blood flow during submaximal and maximal dynamic exercise in humans. J. Appl. Physiol.

[b53] Van Lieshout JJ, Pott F, Madsen PL, van Goudoever J, Secher NH (2001). Muscle tensing during standing: effects on cerebral tissue oxygenation and cerebral artery blood velocity. Stroke.

[b54] Verbree J, Bronzwaer AS, Ghariq E, Versluis MJ, Daemen MJ, van Buchem MA (2014). Assessment of middle cerebral artery diameter during hypocapnia and hypercapnia in humans using ultra-high-field MRI. J. Appl. Physiol.

[b55] Wieling W, Low PA, Van Lieshout JJ (1997). Maintenance of postural normotension in humans. Clinical autonomic disorders.

